# Electronic signature and attestation in conveyancing practice: A Malaysian legal perspective

**DOI:** 10.12688/f1000research.73548.3

**Published:** 2022-12-05

**Authors:** Hua Siong Wong, Mohd Munzil Muhamad

**Affiliations:** 1 Faculty of Law, Multimedia University, Jalan Ayer Keroh Lama, Melaka, 75450, Malaysia

**Keywords:** Attestation, Conveyancing Practice, Electronic Signature, Malaysia.

## Abstract

Background

The Corona Virus Disease 2019 (COVID-19) pandemic brought about an unprecedented disruption to global business activities. Physical face-to-face activities must be restricted due to movement control order (MCO). The clients are required to sign the documents physically in the presence of the solicitor who must subsequently attest to the signature of the clients. The issue arises whether electronic signature (e-signature) and attestation are permissible under the laws of Malaysia. The aim of this research was to study the legality of e-signature and attestation in conveyancing practice in Malaysia and subsequently to propose recommendations to overcome these issues.

Methods

This is qualitative study and not an empirical study. The data was collected by library-based research from various primary and secondary data sources, including case law in Malaysia, written statutes, publication of journal and article.

Results

The Digital Signatures Act 1997 (DSA) and the Electronic Commerce Act 2006 (ECA) have legalised e-signatures. The DSA is the law that governs the digital signatures in Malaysia. ECA has listed a few documents which are not legally accepted if signed electronically, namely Power of Attorney, the Wills and codicils, the Trusts, and negotiable instruments. However, with regards to the issue of attestation of these documents, there are no clear laws which govern the attestation. The legal issue arises when the lawyers who have attested these documents are liable to be called as witness under the Evidence Act 1950 to testify their signature if these documents are tendered as evidence in any court proceedings.

Conclusion

Thus, it is suggested that there is a need for unique legal framework for e-signature and attestation in Malaysia due to the lack of specific laws which govern the issues of electronic signature and attestation.

## Introduction

The Corona Virus Disease 2019 (COVID-19) pandemic brought about an unprecedented disruption to the global business activities, including but not limited to the importing and exporting, franchising, joint venture, service activities, i.e., hotel, legal service, retails etc. Due to the movement control order (MCO) and strict standards of procedure (SOP), most of the physical face-to-face activities must be restricted in order to prevent physical contact. In legal line service, especially law firms which handle the property transactions and conveyancing practice, involve a lot of documentation that will require the clients’ signature and subsequently to be attested by the solicitors. However, due to the MCO and that the law firms are not considered as “essential service”, thus, the law firms in West Malaysia are required to temporarily close. It has been suggested by many parties to substitute the physical signatures with electronic signatures (e-signatures) followed by document attestation to be done by video conference calls. In practice, some judges would accept affidavits which have yet to be affirmed subject to solicitors’ undertaking to duly affirm them after the MCO. This scenario can be seen in the case of *SS Precast Sdn. Bhd. Vs. Serba Dinamik Group Bhd. & Ors* [2020] MLJU 400. Due to the MCO, the counsel was not able to affirm the affidavit before a Commissioner for Oaths. In this case, the learned High Court judge Datuk Wong Kian Kheong emphasized that after the MCO, any affirmation of the documents still requires the signatures of the Commissioner for Oaths with wet-ink.

Although e-signature is not a new technology in Malaysia, the issue remains whether e-signature and attestation of the legal documents via video conferencing platform are permissible under the Malaysian laws.

The aim of this research is to study the legality of e-signature and attestation in conveyancing practice in Malaysia. Secondly, the author will identify the legal issues relating to e-signature and attestation and thirdly, the author will propose recommendations to overcome these issues.

## Methods

This study is a qualitative study and adopted doctrinal research methodology. The appropriate data were collected from the library, relevant databases, and other archives. The contents of the current statutory provisions, case law and guidelines on the issues of e-signature and attestation of the documents in the legal practice were analyzed, especially during the Covid-19 pandemic.

This study has explored both primary and secondary data. The primary data included the current statutes, case law, rules and guidelines on e-signature and attestation of the document (See Underlying data) ( Hua Siong, 2021). The relevant articles and journal publication and reference textbooks were categorized as secondary data. Data from library-based research was done in various libraries from local government and private universities. The interviews were performed among the selected respondents.

Meanwhile, this study used content analysis as the main method of data analysis. This method provided an overview of the study and relevant evidence for the facts.

## Results/Discussion

### E-signature in Malaysia

Generally, a signature is proof that the signatory has read and agreed to be bound by the contents of the document. Additionally, it indicates that the signatory has specifically consented to the contents of the documents and wanted it to have a legal effect.

The thumb-print has also been defined as ‘signature’ under the Section 3 of the Interpretation Acts of 1948 and 1967 (Consolidated and Revised 1989) (Act 388) in Malaysia. Under section 210(2) of the National Land Code 1956, a thumb-print on the instrument of dealings is considered legal and valid. However, this type of signature may not serve as conclusive evidence as it may be challenged by other factors, namely duress, undue influence, fraud, forgery etc.

In Malaysia, legislations governing electronic signature include Digital Signature Act 1997 (DSA), Electronic Commerce Act 2006 (ECA) and Electronic Government Activities Act 2007 (EGAA). E-signatures in Malaysia are governed by the Electronic Commerce Act (ECA) 2006. However, for digital signatures, the relevant legislation would be the Digital Signature Act (DSA) 1997.

It is important to understand the differences between digital signatures and e-signatures. Generally, e-signature is not so complex when compared to a digital signature which requires a lot of procedures, such as authentication of public and private keys, digital certificate etc. Under sections 22 to 26 of DSA, these signatures require licenced certification authorities, and such activities can only be performed as specified in the licence. Both signatures are legally recognized in Malaysia by DSA and ECA. Furthermore, for electronic commercial transactions both signatures can be used under the ECA. If digital signature is chosen, then provisions under DSA will be applied. Section 9(1) and (2) of the ECA also recognizes the contracts which have been finalised by the parties in the contracts and were executed electronically, however such contracts and agreements are subject to the Stamp Act. Under the Malaysia Evidence Act 1950, the court accepts and admits any agreements which are signed electronically provided that these documents passed the rules of admissibility of evidence. Under the section 90A of the Evidence Act 1950, it states that the court shall admit the document produced by a computer, or a statement contained in such document as evidence if the document was produced by the computer during its ordinary use, whether the person tendering the same is the maker of such document or statement ( Radhakrishna, 2012).

Meanwhile, the usage of Personal Identification Number (PIN) is in fact permissible under the Prescription of Electronic Signature Order 2010 issued pursuant to the Electronic Government Activities Act 2007. Thus, there is no legal issue about the usage of PIN as electronic signature.

### What is a digital signature?

**Figure 1.**
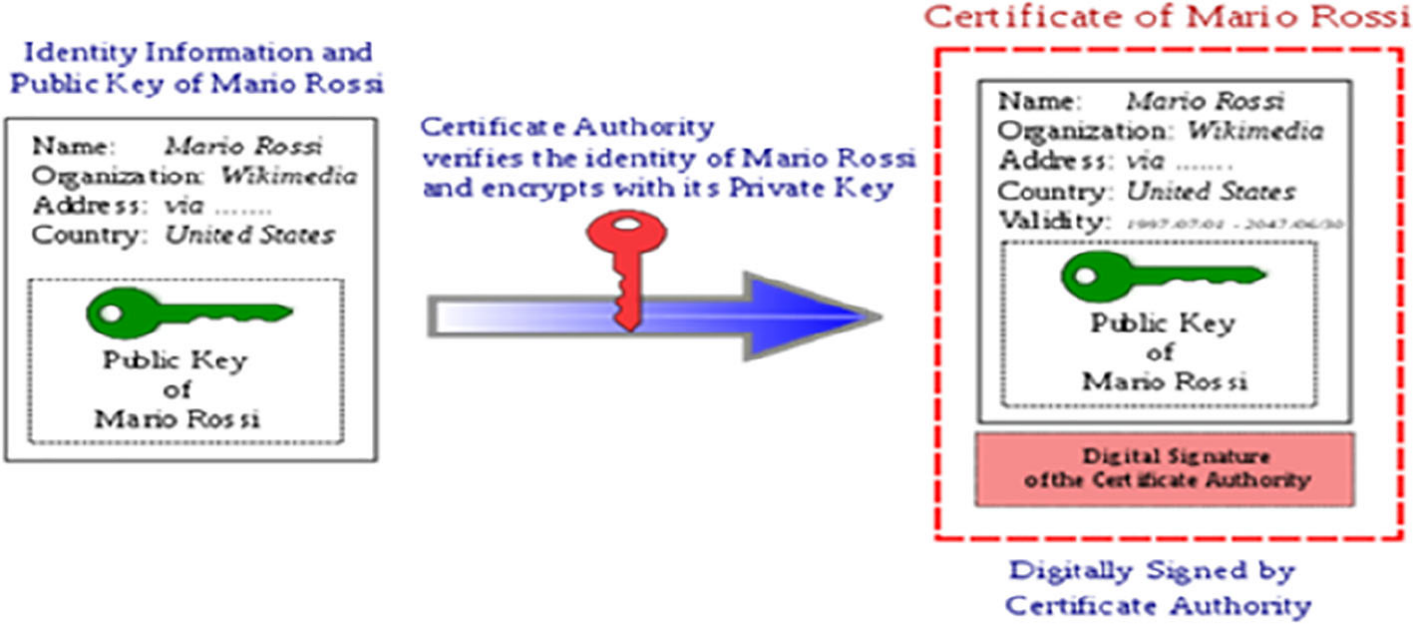
Digital signature and digital signature processing. Source: https://signx.wondershare.com/knowledge/digital-signature-example.html.

According to Nguyen (2013), a digital signature can be defined as one form of e-signature, that is highly safe and widely used. Based on Huang, Chen, and Hoa (2010), digital certificates are an electronic file that can be used as an identity card to verify the identity of a party on the Internet. In order for digital signature to be functionable, it requires a digital certificate which is also known as private key for verification purpose (Kumar, 2019). This private key is only known by the person who is authorised to access the document. The public key held by the owner and creator of the document must correspond with the private key for the purpose of determination of the legitimacy of this digital signature and to track any changes made in the document. Figure 1 briefly explains the process of digital signature.

Digital signature is a good authentication tool as it can provide privacy and high integrity (Jason 2014). Nevertheless, there is still a possibility for a third party to create a false digital signature by using false identity. For example, a hacker can create fake encryption keys and register the public key under the name of individual A. The hacker can use the fake private key of individual A to encrypt a message and sent it to individual B. Upon receiving the message, individual B will think that it is a genuine message from individual A. However, as the content of the message is not correct, it will mislead individual B, or it may even contain harmful executable viral software that could hack the receiver’s computer or system. However, in this paper, we will focus on the issue of e-signature.

### Recognition of E-signature in documents

Malaysia’s Electronic Commerce Act 2006 has adopted the United Nations Commission on International Trade Law (UNCITRAL) Model of Electronic e-signature and as such e-signatures are accepted and recognized worldwide. This practice has become more popular among corporate transactions, including the commercial agreements between corporate entities and due diligence documents for corporate proposals which involve transactions of more than million US dollars. However, the security issue of e-signatures is always a priority to e-signature users.

Part III of the ECA recognizes that e-signatures are legally enforceable provided that the following requirements are fulfilled including but not limited to (I) satisfying the definition of an e-signature in ECA; (II) the signature being logically associated with the electronic document; (III) to be able to identify the signature of the owner and obtain the approval of the information in the document; (IV) the signature to be under control of the owner; and (V) any changes in the signature or the associated documents to be detectable.

The ECA has listed few documents which cannot use e-signature in the same effect due to the security and trust issues, these documents include but are not limited to Power of Attorney, the creation of Wills and Codicils, the creation of Trusts, and negotiable instruments, particularly in banking documents. Another reason that these documents in specific Wills and Codicils, Trusts are not allowed to use e-signatures is to prevent fraudulent activities from the third parties as these are based on the intention of the makers.

Meanwhile, some commercial documents, namely company’s annual accounts, share transfer instruments, and documents from the court may require formal attestation from the Commissioner for Oaths or notarization by a Notary Public or a seal to be affixed under any other laws, i.e., Companies Act 2016, as such e-signatures will not be suitable.

To date, there were not many cases that deliberated on the application of the ECA. However, the recent case of *Yam Kong Seng & Anor v Yee Weng Kai* [2014] 4 MLRA 316 does illustrate the point above that it is not necessarily a very difficult threshold to meet in order to identify the signatory. In that case, the court has agreed to accept even a simple short message service (SMS) to be considered as fulfillment of the legal requirement for a signature. It is important to note that s.66(4) of the Malaysia Companies Act 2016 (“CA 2016”) states that it is not compulsory to use common seal for a company’s document as it can also be signed by an authorized officer. If the authorized officer can sign on behalf of the company, it is safe to argue that the officer’s e-signature may also be accepted provided the requirements under the ECA are fulfilled.

It is important to note that the reliability of a document, for example trade commercial agreements which use an e-signature, could still be challenged in court. This is because the e-signature is just a symbol affixed to an electronic document, it is impossible to track the changes made to the document at the time of signature. At the moment, there aren’t any tools that can verify the identity of the person, or whether the document was signed voluntarily under duress conditions. To reduce the possibility of authentication being raised, all these practical difficulties should be addressed.

### Can A conveyancing solicitor attest the signing of a document via video conferencing platform?

Due to restriction on in person meetings, it is important to understand whether a conveyancing solicitor can attest the signing of a document via video conferencing platform, namely Zoom or Google Meet. In regard to this issue, the Malaysian law pertaining to the attestation of the documents or witnessing a signature via video conferencing is still very uncertain and silent to whether they legally recognize this kind of process. As Malaysian courts have started to transition to online hearings gradually and steadily ( Jeyakuhan, 2020), Bar Council Malaysia has issued their latest Circular No. 222/2021 to clarify the issue of attestation of the signing of the documents via video conferencing platform. Pursuant to this Circular, the solicitors are advised to seek a third-party legal advice before proceedings. However, the Circular is not clear as to whether the solicitors are permitted to attest the signing of the documents via video conferencing platform. In 2022, Bar Council Malaysia has re-issued a new Circular No. 218/2022 pertaining to the new ruling 14.11(2) to set the procedure for virtual or remote attestation or witnessing of execution of a document by a solicitor. The main purpose for the presence of a solicitor is to witness and attest signing of a document is to prove the identity of the signature and to make sure that the document is getting signed voluntarily and not under the duress. Section 15 of the Malaysia Contracts Act 1950 defined the meaning of coercion. In the case of *Kanhaya Lal v National Bank of India Ltd* (1913) 40 Ind App 56 (PC) 83, the Privy Council explained that in section 15, the term “coercion” refers to an illegal conduct committed “with the goal of inducing the person to enter into an agreement.” Thus, prior to the existence of the contract, the parties must give consent to be bound by the promise and this consent must, however, be obtained freely without any pressure from the other party. Meanwhile, if the solicitor attests a signing ceremony via video conferencing, they would not be able to confirm that the signatory is not under any duress from the third party who might be present in the room but not visible on the video conference call.

The next issue is understanding the definition of “in the presence of”’ and whether it/should be given a strict or wide interpretation. Unfortunately, the laws in Malaysia are still unclear about this definition. There is an issue of whether watching someone signing a document via video conferencing would amount to “signing in the presence of this person” although both the signatory and the person who witnessed the signing were not in the same room. Besides that, it is also difficult for the witness (Solicitor) to confirm that the document that is subsequently sent to him for attestation is the same document he saw on the screen.

## Conclusions

In light of Covid-19 outbreak and social distancing, legislators will likely need to review and amend existing laws to cater for current situations during the pandemic. In essence, e-signatures can work as substitute if face-to-face meetings are not practicable for signature purpose. However, we need to be very caution about the usage of e-signatures to avoid any misuse of its convenience. Attestation via video conferencing needs to be addressed clearly by taking into consideration the meaning of ‘in the presence of’ as the laws in Malaysia are unclear about this issue. In order to prevent any fraudulent activities, Malaysia has also started the biometric system, especially in the dealing with land trades for collection of original land title which takes place at the relevant land office/registry. However, it needs to be considered that it is strongly advisable to have the documents to be signed physically to avoid any risk of dispute about the authenticity of the execution of the documents.

In Malaysia, it has become a practice that all the government departments for example land office and stamping office will only accept relevant documents to be signed in black fountain wet-ink. These departments do not accept any electronic e-signatures. This practice is to avoid any fraudulent transactions as the land office, including the Registrar of Titles, has a duty of care toward proprietors of the land to safeguard their interest, as indicated by cases *Pendaftar Hakmilik Negeri Selangor & Ors v Shaifulizam Mohd Saleh & Anor And Another Appeal* [2020] 5 CLJ 595 and *Pendaftar Hakmilik Negeri Selangor v Caesius Development Sdn Bhd & Ors And Another Appeal* [2020] 3 CLJ 327.

Perhaps it can be beneficial for the current ECA to be amended to allow conveyancing solicitors to attest the signing of a document via video conferencing platform. Parties are thus advised to seek independent legal advice from those who are experts in this area of law to protect their rights.

## Data availability

### Underlying data

Figshare: Electronic signature and attestation in conveyancing practice: A Malaysian legal perspective https://figshare.com/articles/dataset/Cases_zip/16530738 ( Hua Siong, 2021).

This project contains the following underlying data:

Case 1-5. The case studies and analyses mentioned in this study.

Data are available under the terms of the Creative Commons Zero “No rights reserved” data waiver Attribution 4.0 International (CC BY 4.0)

## Author contributions

Conceptualization, Data Curation, Funding Acquisition, Project Administration, Writing – Original Draft Preparation, by Wong Hua Siong. Formal Analysis, Investigation, Methodology, and Resources, Writing – Review & Editing by Wong Hua Siong & Mohd. Munzil Muhamad. Supervision by Mohd. Munzil Muhamad.

## List of statutes

[1] Bar Council Malaysia Circular No 222/2021.

[2] Companies Act 2016.

[3] Contracts Act 1950.

[4] Digital Signature Act 1997.

[5] Electronic Commerce Act 2006.

[6] Electronic Government Activities Act 2007.

[7] The Evidence Act 1950.

[8] The Malaysia Companies Act 2016.
