# Supplementary material for: Electronic signature and attestation in conveyancing practice: A Malaysian legal perspective
Source: F1000Res. 2022 Dec 5;11:325. Originally published 2022 Mar 17. [Version 3] doi: 10.12688/f1000research.73548.3 (PMC10682595; doi:10.12688/f1000research.73548.3)
Supplement: Peer review history

# Reviewer report: Reviewer response for version 3

**DOI:** 10.5256/f1000research.141760.r218977

**Published:** 2023-11-27

**Authors:** Mateja Durovic

**Affiliations:** 1 King's College London, London, England, UK

This is a very well written article, good references, clear structure, original and important topic, excellent legal and critical analysis, adequate methodological approach, clear argumentation, good introduction, main parts and conclusive observations, setting up basis for the future research, it will become an unavoidable piece of literature for anyone doing research in the field

Is the work clearly and accurately presented and does it cite the current literature?

Yes

If applicable, is the statistical analysis and its interpretation appropriate?

Yes

Are all the source data underlying the results available to ensure full reproducibility?

Yes

Is the study design appropriate and is the work technically sound?

Yes

Are the conclusions drawn adequately supported by the results?

Yes

Are sufficient details of methods and analysis provided to allow replication by others?

Yes

Reviewer Expertise:

Law

I confirm that I have read this submission and believe that I have an appropriate level of expertise to confirm that it is of an acceptable scientific standard.

# Reviewer report: Reviewer response for version 3

**DOI:** 10.5256/f1000research.141760.r218986

**Published:** 2023-11-27

**Authors:** Ida Madieha Abd Ghani Azmi

**Affiliations:** 1 Ahmad Ibrahim Kulliyyah of Laws, International Islamic University Malaysia, Kuala Lumpur, Federal Territory of Kuala Lumpur, Malaysia

The author could have investigated the practice in other countries eg UK and Australia on the acceptability of document attestation using video conference. Surely, practices done during Covid are only temporary measures and cannot be the general rule, especially for legal documents. Perhaps the author can explore further what other countries are practising re attestation of legal documents.

Is the work clearly and accurately presented and does it cite the current literature?

Yes

If applicable, is the statistical analysis and its interpretation appropriate?

Not applicable

Are all the source data underlying the results available to ensure full reproducibility?

Yes

Is the study design appropriate and is the work technically sound?

Partly

Are the conclusions drawn adequately supported by the results?

Yes

Are sufficient details of methods and analysis provided to allow replication by others?

Yes

Reviewer Expertise:

intellectual property law, cyber law

I confirm that I have read this submission and believe that I have an appropriate level of expertise to confirm that it is of an acceptable scientific standard, however I have significant reservations, as outlined above.

# Reviewer report: Reviewer response for version 1

**DOI:** 10.5256/f1000research.77206.r139343

**Published:** 2022-06-20

**Authors:** Noraziah Abu Bakar

**Affiliations:** 1 Faculty of Law, Universiti Teknologi MARA, Selangor, Malaysia

The paper is an excellent and essential addition to the literature. However, the discussion on the attestation of instrument of dealings prescribed under the conveyancing practices in Malaysia is regulated under the National Land Code (Revised 2020) (Act 828) where it is strictly regulated by Act 828 that attestation by lawyers shall comply with Section 211 and 5th Schedule of the Act. Thus, when execution of the instrument is dealing is done by a natural person, a lawyer shall attest to the signature of the parties and sign the attestation clause provided in the instrument. The attestation indicates that the execution (signing) is done in the presence of the lawyer. The conclusions are well written but it is silent on the impact of e-signature under the abovementioned provisions of Act 828.

Is the work clearly and accurately presented and does it cite the current literature?

Yes

If applicable, is the statistical analysis and its interpretation appropriate?

Yes

Are all the source data underlying the results available to ensure full reproducibility?

Yes

Is the study design appropriate and is the work technically sound?

Yes

Are the conclusions drawn adequately supported by the results?

Yes

Are sufficient details of methods and analysis provided to allow replication by others?

Yes

Reviewer Expertise:

Land Law and Conveyancing Practice in Malaysia.

I confirm that I have read this submission and believe that I have an appropriate level of expertise to confirm that it is of an acceptable scientific standard.

# Reviewer report: Reviewer response for version 1

**DOI:** 10.5256/f1000research.77206.r130205

**Published:** 2022-04-28

**Authors:** Rod Thomas

**Affiliations:** 1 Law and Commerce, AUT University, Auckland, New Zealand

The authors are to be commended for raising a practical issue which needs resolution in the “Time of Covid”. I take this to be the need to satisfactorily prove the witnessing of conveyancing documents when, under covid conditions, person-to-person meetings are not permissible.

 The article opens with a broad discussion of legal issues related to signatures and attestation. The authors recognise that, in essence, a signature is some physical “affixing” to a document showing an intention to be bound. They acknowledge that, at law, a signature may be an inked thumb print, an “e-signature” or even a “digital signature”. They also record the purpose of the Digital Signatures Act 1997 (DSA) and the Electronic Commerce Act 2006 (ECA) which, in combination, may enable a “digital” signature to be accepted for evidential purposes, providing certain statutory protocols are adhered to. The article then extends on to mention to other issues in practice, such as some government agencies only accepting signatures made in “black fountain wet-ink”. The authors reflect that, welcome as the provisions in the DSA and ECA are, those provisions do not adequately extend to situations where execution must be undertaken before a witness who is not physically present under covid conditions.

 This last point I take to be the central issue that the article ultimately seeks to address, although this is not clear because of the preceding, rather general discussion. The question then posed, appears to be whether execution of conveyancing documents can be understood to be “before” a witness who is only available by an electronic link. The conclusion in the abstract is representative of the broad ranging discussion that has occurred. It reads as follows.

 “Thus, it is suggested that there is a need for a unique legal framework for e-signature and attestation in Malaysia due to the lack of specific laws which govern the issue of electronic signature and attestation”.

 It seems to me that the article would have benefited from isolating out, and then starting with the key issue to be addressed.  As stated, I take this to be the present need for conveyancing documents to be signed before witnesses who are physically present. The material presented could then be better positioned to discuss possible solutions. Mention of the requirements of the DSA and ECA do have their place, but only in the sense of identifying missed opportunities.

 By the time I got to the conclusion, I was made aware of the problem, but was unclear on the solution. On the one hand the authors seem to suggest “e-signatures are a viable solution”, notwithstanding their recognition that for e-signatures, “safety factors” remain a concern.  At the same time they suggest the ECA be “amended to allow conveyancing solicitors to attest the signing of a document via video conferencing platform[s]”.

 In essence, I was left a little uncertain whether the purpose of the article was to identify all legal issues related to electronic signatures, or to discuss  possible solutions for signatures to conveyancing documents that must be signed before a witness. The two topics are interrelated, but different.

 The authors are to be commended for taking the first step into the arena. We all benefit from a discussion such as this.

Is the work clearly and accurately presented and does it cite the current literature?

Partly

If applicable, is the statistical analysis and its interpretation appropriate?

Not applicable

Are all the source data underlying the results available to ensure full reproducibility?

Yes

Is the study design appropriate and is the work technically sound?

Yes

Are the conclusions drawn adequately supported by the results?

Partly

Are sufficient details of methods and analysis provided to allow replication by others?

Yes

Reviewer Expertise:

Real property, sale of land, high density living, property law, equity, art law

I confirm that I have read this submission and believe that I have an appropriate level of expertise to confirm that it is of an acceptable scientific standard, however I have significant reservations, as outlined above.

# Response

**Published:** 2022-07-21

**Authors:** Wong Hua Siong

**Affiliations:** Multimedia University, Malaysia

Thank you for the feedback and will incorporate the comments and improve the contents accordingly.
